# Rietveld in 100 picoseconds

**DOI:** 10.1107/S1600576721000704

**Published:** 2021-02-01

**Authors:** Brian H. Toby

**Affiliations:** a Argonne National Laboratory, 9700 South Cass Avenue, IL 60439, USA

**Keywords:** powder diffraction, pink beams, Rietveld refinement

## Abstract

A recent article by Von Dreele, Clarke & Walsh [*J. Appl. Cryst.* (2021), **54**, https://doi.org/10.1107/S1600576720014624] introduces an entirely new paradigm in structure determination, where a complete structural measurement is made in a tenth of a nanosecond.

For those of us of a certain age, collection of a data set for a crystal structure represents a major undertaking. With a single crystal and a laboratory instrument, this now requires hours, rather than days or even the weeks of my youth, and is even slightly faster with a synchrotron. From a powder sample, a diffraction data set now typically requires at least minutes and perhaps hours in the laboratory, but can be completed in a second with an area detector at a synchrotron. The structure determined is an average over the measurement duration. But an entirely new paradigm in structure determination, where a complete structural measurement is made in a tenth of a nanosecond, is demonstrated in this issue of the journal by Robert B. Von Dreele, Samantha Clarke and James Walsh in their article *‘Pink’-beam X-ray powder diffraction profile and its use in Rietveld refinement* ▸. This is truly a new age.

To understand how this works, one must know a bit about the functioning of a synchrotron. Diffractionists tend to think of a synchrotron as a constant source of X-rays, but just as the screen you are reading this on is actually flashing at something like 60 or 120 images each second – faster than our eyes can recognize – the X-rays generated from a synchrotron flash on only while a packet of electrons travels past a bending magnet or insertion device at 99.99…% of the speed of light. The X-rays from this pulse illuminate the sample for only *ca* 0.1 ns. The synchrotron storage ring is filled with a relatively small number of electron packets, so there is a delay of 10–200 ns before the next pulse arrives. The electron pulses circulate around the storage ring and return to the same location in a few microseconds so the flashes repeat in a regular pattern. For most experiments, the on/mostly-off flashing of the synchrotron X-rays is not perceptible, no more than the much slower blinking of our room lights and computer screens. However, it is possible to use high-speed choppers and detector gating to isolate the X-rays scattered from a single electron pulse as an image on an area detector. In a normal synchrotron diffraction instrument, a perfect-crystal monochromator allows only an extremely narrow band of X-ray wavelengths to reach the sample, so few X-rays arrive at the sample and thus far too few are scattered from that pulse to be useful in a single measurement. However, at a small number of beamlines, such as the APS Dynamic Compression Sector (DCS; https://dcs-aps.wsu.edu) beamlines, the wavelength band is greatly expanded to produce what is called a pink beam, and this allows for a much larger number of photons to be measured from the diffraction of a single synchrotron pulse.

The X-ray spectrum from the DCS pink-beam monochromator is somewhat complex and one might expect this would limit the analysis of diffraction data sets only to a qualitative level of analysis. However, Von Dreele *et al.* (2021 ▸) show that quantitative Rietveld (1969 ▸) fitting to these data is possible, allowing full structural determination up to the limits allowed by the relatively poor peak resolution and signal-to-noise ratio offered by the technique. Since the X-rays are of fairly high energy, the spatial resolution can be atomistic. Peak resolution and sensitivity will tend to limit the complexity of models that can be developed, but working around this is not a new problem in powder diffraction (*International Tables for Crystallography*, 2019 ▸).

Note that this work was greatly facilitated by one author’s pioneering experience in developing analysis software for pulsed (time-of-flight, TOF) neutron diffraction (Von Dreele *et al.*, 1982 ▸), which he noticed offers a similar energy spectrum, even though it arises from a completely different physical process. This demonstrates the value of scientific breadth. No one could have expected that TOF peak shapes would ever be needed at a synchrotron.

While I am far from impartial on this subject, I would also comment that this work points to the value of modern software projects such as *GSAS-II* (Toby & Von Dreele, 2013 ▸) that encompass an extended scope and are written in modern languages. While I am certain that analysis of pink-beam diffraction data was not contemplated in the initial design of the package (Toby & Von Dreele, 2007 ▸), the flexible data structures provided by Python and the open framework of the project made it a relatively easy task to support a new experiment type and its associated peak shape, despite not being part of the initial project design. This probably would have been a much more difficult task in an old-style Fortran project, such as *GSAS* (Larson & Von Dreele, 2004 ▸).

From a diffraction pattern of a state that exists for only 0.1 ns, one can imagine probing the atomistic structure of many exotic transitory chemical and physical states that might be produced in a bulk material. The synchrotron then becomes a stroboscope that freezes ultrafast motion. The interests that prompted construction of the DCS beamlines via funding from the US National Nuclear Security Administration are largely associated with the science of transient pressure spikes in a wide variety of materials, but one can also imagine a range of chemical and physical probes that will produce a rapid structural response. The work of Von Dreele *et al.* here shows that it is now possible to determine structures quantitatively under these conditions. This truly opens new frontiers for structural science. Even faster sampling is possible with free-electron X-ray lasers (FELs), but in more than a decade of FEL sources, there have been very few powder diffraction structural studies (Blome *et al.*, 2005 ▸; Freyer *et al.*, 2013 ▸; Wehrenberg *et al.*, 2017 ▸). Perhaps this work will encourage more.
